# Neurofunctional reorganization and memory improvements induced by rivastigmine in Alzheimer's disease are mediated by α4β2 nicotinic receptor density

**DOI:** 10.1002/alz70856_102662

**Published:** 2025-12-24

**Authors:** Riccardo Manca, Matteo De Marco, Annalena Venneri

**Affiliations:** ^1^ University of Parma, Parma, Italy; ^2^ Brunel University London, Uxbridge, Middlesex, United Kingdom; ^3^ Brunel University London, London, United Kingdom; ^4^ University of Parma, Parma, Italy, Italy

## Abstract

**Background:**

Acetylcholinesterase inhibitors are used to delay cognitive decline due to Alzheimer's disease (AD) by preventing breakdown of acetylcholine. Such treatment appears to be associated with cerebral changes, although the mechanism of action has not been clarified yet. This study aimed at assessing rivastigmine‐induced changes in acetylcholine‐related functional connectivity (FC) and cognitive performance in patients with prodromal to mild AD.

**Method:**

Thirty‐two patients with prodromal to mild AD recruited consecutively from a memory clinic were randomly assigned either to a 12‐week rivastigmine patch treatment (ADt; *n* = 16) or to an untreated control group (ADu; *n* = 16). Participants underwent comprehensive neuropsychological and MRI assessments at baseline (T0) and after 12 weeks (T1). Functional MRI scans were pre‐processed with the REACT pipeline to extract whole‐brain FC maps weighted by the expected distribution of the vesicular acetylcholine transporter, the α4β2 nicotinic and the M1 muscarinic receptors using publicly available atlases. Between‐group differences in T0‐to‐T1changes in cognitive performance and neurotransmitter‐related FC were investigated. A regression model was used to assess the association between longitudinal changes (T1‐T0) in cognitive performance and FC in the ADt group.

**Result:**

At T0, the ADt group had worse visual long‐term memory and higher α4β2‐related FC in frontal and occipito‐cerebellar cortices compared with the ADu group. Compared with the ADu group, the ADt group showed a greater longitudinal reduction in frontal α4β2‐related FC and a greater improvement in visual memory. Changes in α4β2‐related FC were significantly associated with memory improvements.

**Conclusion:**

Rivastigmine treatment induced FC changes in frontal and occipito‐cerebellar areas in patients with prodromal to mild AD that were associated with long‐term memory performance improvements over 12 weeks. These rivastigmine‐induced effects on resting‐state brain activity and the mediating role of the α4β2 nicotinic receptor for acetylcholine are in line with previous findings. Neurotransmitter‐related FC can be an MRI biomarker sensitive to test mechanisms of actions and to track neuroplastic changes induced by pharmacological treatments targeting specific neurotransmitter systems in people with AD.

